# Improved Alzheimer Disease Diagnosis With a Machine Learning Approach and Neuroimaging: Case Study Development

**DOI:** 10.2196/60866

**Published:** 2025-04-21

**Authors:** Lilia Lazli

**Affiliations:** 1 Department of Computer and Software Engineering, Polytechnique Montréal, University of Montreal, 2500 Chem de Polytechnique, Montreal, QC, H3T 1J4, Canada, 1(514) 340-5121 ext 3750

**Keywords:** Alzheimer disease, computer-aided diagnosis system, machine learning, principal component analysis, linear discriminant analysis, t-distributed stochastic neighbor embedding, feedforward neural network, vision transformer architecture, support vector machines, magnetic resonance imaging, positron emission tomography imaging, Open Access Series of Imaging Studies, Alzheimer's Disease Neuroimaging Initiative, OASIS, ADNI

## Abstract

**Background:**

Alzheimer disease (AD) is a severe neurological brain disorder. While not curable, earlier detection can help improve symptoms substantially. Machine learning (ML) models are popular and well suited for medical image processing tasks such as computer-aided diagnosis. These techniques can improve the process for an accurate diagnosis of AD.

**Objective:**

In this paper, a complete computer-aided diagnosis system for the diagnosis of AD has been presented. We investigate the performance of some of the most used ML techniques for AD detection and classification using neuroimages from the Open Access Series of Imaging Studies (OASIS) and Alzheimer’s Disease Neuroimaging Initiative (ADNI) datasets.

**Methods:**

The system uses artificial neural networks (ANNs) and support vector machines (SVMs) as classifiers, and dimensionality reduction techniques as feature extractors. To retrieve features from the neuroimages, we used principal component analysis (PCA), linear discriminant analysis, and t-distributed stochastic neighbor embedding. These features are fed into feedforward neural networks (FFNNs) and SVM-based ML classifiers. Furthermore, we applied the vision transformer (ViT)–based ANNs in conjunction with data augmentation to distinguish patients with AD from healthy controls.

**Results:**

Experiments were performed on magnetic resonance imaging and positron emission tomography scans. The OASIS dataset included a total of 300 patients, while the ADNI dataset included 231 patients. For OASIS, 90 (30%) patients were healthy and 210 (70%) were severely impaired by AD. Likewise for the ADNI database, a total of 149 (64.5%) patients with AD were detected and 82 (35.5%) patients were used as healthy controls. An important difference was established between healthy patients and patients with AD (*P*=.02). We examined the effectiveness of the three feature extractors and classifiers using 5-fold cross-validation and confusion matrix–based standard classification metrics, namely, accuracy, sensitivity, specificity, precision, *F*_1_-score, and area under the receiver operating characteristic curve (AUROC). Compared with the state-of-the-art performing methods, the success rate was satisfactory for all the created ML models, but SVM and FFNN performed best with the PCA extractor, while the ViT classifier performed best with more data. The data augmentation/ViT approach worked better overall, achieving accuracies of 93.2% (sensitivity=87.2, specificity=90.5, precision=87.6, *F*_1_-score=88.7, and AUROC=92) for OASIS and 90.4% (sensitivity=85.4, specificity=88.6, precision=86.9, *F*_1_-score=88, and AUROC=90) for ADNI.

**Conclusions:**

Effective ML models using neuroimaging data could help physicians working on AD diagnosis and will assist them in prescribing timely treatment to patients with AD. Good results were obtained on the OASIS and ADNI datasets with all the proposed classifiers, namely, SVM, FFNN, and ViTs. However, the results show that the ViT model is much better at predicting AD than the other models when a sufficient amount of data are available to perform the training. This highlights that the data augmentation process could impact the overall performance of the ViT model.

## Introduction

Alzheimer disease (AD) is a progressive degenerative brain disorder that gradually destroys memory, reason, judgment, language, and ultimately the ability to perform even the simplest of tasks [1]. An automated AD classification system is crucial for the early detection of disease. This computer-aided diagnosis (CAD) system can help expert clinicians prescribe the proper treatment and prevent brain tissue damage [1].

In the last decades, researchers have developed several CAD systems [1-5]. Rule-based expert systems were developed from the 1970s to the 1990s and supervised models from the 1990s [1]. Moreover, several approaches have been proposed in the literature aiming at providing an automatic tool that guides the clinician in the AD diagnosis process [1,5-7]. We can categorize these approaches into two types: univariate approaches, like statistical parametric mapping (SPM), and multivariate approaches, like the voxels-as-features (VAF) approach.

Due to advances in computing power, machine learning (ML) has encompassed many health care sectors and has shown results with organ and substructure segmentation as well as disease classifications in areas of pathology, brain, breast, bone, retina, etc. Open-access datasets on AD have led to the development of CAD systems that use ML to help scientists and medical staff make early diagnoses. These systems will ultimately help speed up the treatment of patients with AD. To make predictions, scientists have adopted various ML-based classifiers, including support vector machines (SVMs) [8,9], hidden Markov models [10,11], *k*-nearest neighbors classifier [12,13], discriminant analysis [14,15], random forest [16,17], decision trees [18], naive Bayes classifier [19,20], and artificial neural networks (ANNs) [21,22].

Despite the efforts of researchers, there have been few works on AD detection using ML models that have had significant performance, and the development of an automated AD classification model remains a rather challenging task. Within this framework of distinguishing between healthy controls (HCs) and people with AD, the main contributions of this paper can be summarized as follows.

- We developed a CAD system using the best-supervised learning classifiers, such as SVMs [8,9], feedforward neural networks (FFNNs) [23], and transformer neural networks, especially the vision transformer (ViT) architecture [24], which is becoming more popular in the field of computer vision due to its effectiveness.
- We designed these models to analyze the two neuroimages commonly used in AD diagnosis, namely, structural magnetic resonance imaging (sMRI) and fluorodeoxyglucose (FDG)–positron emission tomography (PET) as these modalities are the preeminent sources of information in the CAD process.
- The multimodal CAD system uses principal component analysis (PCA) [25] in conjunction with SVM and FFNN, training them on the PCA features extracted from the neurological images.
- The most challenging datasets, namely the Open Access Series of Imaging Studies (OASIS) [26] and Alzheimer’s Disease Neuroimaging Initiative (ADNI) [27] datasets, underwent rigorous tests using various experimental settings. These experiments validated the effectiveness of the chosen models, showcasing their superiority over state-of-the-art approaches in terms of accuracy, sensitivity, specificity, precision, *F*_1_-score, and area under the receiver operating characteristic curve (AUROC).

## Methods

### Participants

Sometimes we found signs of AD in the brain data of healthy and older patients, so considerable experience and knowledge were essential to distinguish the AD data from the HC patients’ data. In this context, we have experimented the performance of the proposed CAD system on the OASIS [26] and ADNI [27] datasets.

#### OASIS Dataset

The OASIS dataset [26] was prepared by Dr Randy Buckner from the Howard Hughes Medical Institute at Harvard University, the Neuroinformatics Research Group at Washington University School of Medicine, and the Biomedical Informatics Research Network. OASIS is a longitudinal multimodal neuroimaging, clinical, cognitive, and biomarker dataset for normal aging and AD. We selected the patients with and without dementia from a larger database and obtained them from the longitudinal pool of the Washington University Alzheimer Disease Research Center. The experiment used a dataset that included 90 cognitively normal patients and 210 individuals with AD. The AD group included very mild, mild, moderate, and severe dementia.

#### ADNI Dataset

The ADNI dataset [27], which is the most commonly used in machine learning tasks, is an association of medical centers and universities located in the United States and Canada. ADNI is funded by the National Institute on Aging and the National Institute of Biomedical Imaging and Bioengineering, and through generous contributions from the following: AbbVie; Alzheimer’s Association; Alzheimer’s Drug Discovery Foundation; Araclon Biotech; BioClinica, Inc; Biogen; Bristol-Myers Squibb Company; CereSpir, Inc; Cogstate; Eisai Co., Ltd; Elan Pharmaceuticals, Inc; Eli Lilly and Company; EUROIMMUN; F. Hoffmann-La Roche Ltd and its affiliated company Genentech, Inc; Fujirebio; GE HealthCare; IXICO plc; Janssen Alzheimer Immunotherapy Research & Development, LLC; Johnson & Johnson Pharmaceutical Research & Development, LLC; Lumosity; Lundbeck; Merck & Co., Inc; Meso Scale Diagnostics LLC; NeuroRx Research; Neurotrack Technologies; Novartis Pharmaceuticals Corporation; Pfizer Inc; Piramal Imaging; Servier; Takeda Pharmaceutical Company; and Transition Therapeutics. The Canadian Institutes of Health Research is providing funds to support ADNI clinical sites in Canada. Private sector contributions are facilitated by the Foundation for the National Institutes of Health. The grantee organization is the Northern California Institute for Research and Education, and the study is coordinated by the Alzheimer’s Therapeutic Research Institute at the University of Southern California. ADNI data are disseminated by the Laboratory for Neuroimaging at the University of Southern California.

The main aim of ADNI is to provide open-source datasets to discover biomarkers and identify and track the progression of AD accurately. It developed to become an ideal source of longitudinal multisite PET and magnetic resonance imaging (MRI) images of patients with AD and older control patients (HC). The datasets were formed to make the detection system powerful by providing baseline information regarding changes in brain structure and metabolism, as well as clinical, cognitive, and biochemical data. The ADNI cohort used in our study included 82 cognitively normal patients and 149 patients with AD. The AD group included patients with mild cognitive impairment and those with confirmed AD.

### Ethical Considerations

This work used two datasets (ADNI and OASIS), which are available in the public domain. For the benchmark ADNI dataset, the terms of use are declared on their website [28]. All patients in the ADNI database provided written informed consent, which was approved by the institutional review board of each participating institution. Patients were informed that their information would be kept confidential and their data would be anonymous and would be part of scientific publications.

According to local legislation and institutional requirements, the study of human participants using the OASIS dataset does not require ethical review and approval [26]. Written informed consent from the patients’ legal guardians or next of kin was not required to participate in this study in accordance with national legislation and institutional requirements [26]. The data used for the analysis has been deidentified and made public.

### Data Preparation

We performed the following steps on the OASIS and ADNI neuroimages: normalization, resizing, removing nonbrain slices, selecting slices with the most information, and converting 3D images into 2D slices. First, the damaged original files containing the images were removed. We selected a larger number of central slices to aid the CAD system in accurately classifying AD. We used an SPM tool (SPM8 [29]), which is a major update to SPM software, originally developed by Karl Friston, to partially correct spatial intensity inhomogeneities. This software normalized all the images using a general affine model with 12 parameters. The origin of the raw sMRI scans was set manually to anterior commissure before manually registering them with SPM’s canonical T1 template image. We applied the nonparametric nonuniform intensity normalization (N3) technique to solve the tissue intensity nonuniformity problem [30]. Then the hybrid median filter was used to remove impulse noise while preserving edges.

### ML Approaches

#### Overview

**Figure 1.**
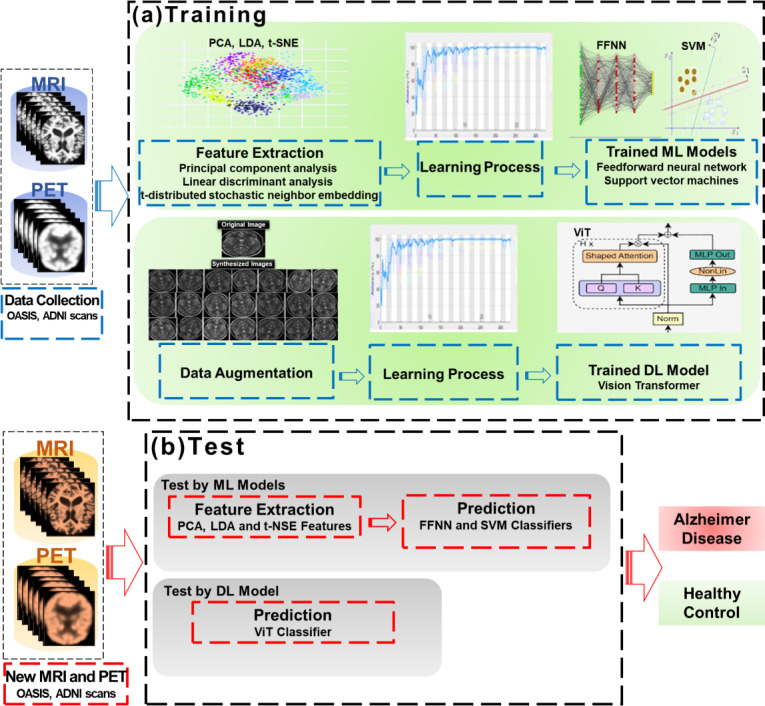
Block diagram of a generic Alzheimer disease computer-aided diagnosis system. ADNI: Alzheimer’s Disease Neuroimaging Initiative; DL: deep learning; FFNN: feedforward neural network; LDA: linear discriminant analysis; ML: machine learning; MRI: magnetic resonance imaging; OASIS: Open Access Series of Imaging Studies; PCA: principal component analysis; PET: positron emission tomography; SVM: support vector machine; t-SNE: t-distributed stochastic neighbor embedding; ViT: vision transformer.

A generic automated AD detection and classification framework is summarized in Figure 1. ML classifiers aim to predict the class of the input data (images of patients with AD or healthy patients) by looking at a number of learning examples. The process begins with the preprocessing of sMRI and FDG-PET images to keep only relevant data. Then each image is represented by grayscale features and is collapsed into a new feature space by applying PCA-based feature extraction to pick the optimal features. After that, to classify the patients, these selected features are fed to the supervised learner. In this work, SVMs and FFNNs are learned on the PCA features extracted from the neuroimages. While for ViT, we applied the data augmentation strategy [31], since the training of this network required more data compared to the other two classifiers. For PCA, a performance comparison was made with similar techniques, t-distributed stochastic neighbor embedding (t-SNE) [32] and linear discriminant analysis (LDA) [14].

Below is a summary description of the four approaches proposed for our CAD system, and more details on the mathematical background of these approaches can be found in Multimedia Appendix 1 for PCA, Multimedia Appendix 2 for SVM, Multimedia Appendix 3 for FFNN, and Multimedia Appendix 4 for ViT.

#### Principal Component Analysis

PCA is a linear dimensionality reduction method used widely in data preprocessing and exploratory analysis. Different image classification purposes have successfully used PCA because its method is nonparametric and easy to apply, and helps extract useful information from confusing datasets [25].

In this study, we used this technique to extract useful features for classifiers. PCA allows the production of new variables that represent linear combinations of the original variables. Using linear algebra and matrix operations, a transformation is performed from the original dataset to a new coordinate system structured by the principal components. The analysis of this linear transformation is obtained thanks to the eigenvectors and the eigenvalues of the covariance matrix. The PCA steps are summarized as follows: (1) standardize the range of continuous initial variables, (2) find correlations by computing the covariance matrix, (3) find the eigenvectors and eigenvalues of the covariance matrix, (4) choose the principal components, and (5) change the data to the new coordinate system. More details about the PCA computation process with mathematical formulas are explained in Multimedia Appendix 1.

#### Support Vector Machines

We used SVMs as classifiers for the classification of independent and identically distributed data [23]. These machines are widely used as supervised max-margin models, along with associated learning algorithms that analyze data. To distinguish two classes, the principle of SVMs is to seek the optimal hyperplane that allows for maximizing the margin between the closest data points of the opposite classes.

The SVM algorithm for linear classification is widely used in ML. However, in this study, we used SVMs to perform nonlinear classification due to the data’s nonlinear separability. We achieved this by applying a kernel function to represent the data as a set of pairwise similarity comparisons between the original data points.

This function transforms the original data points into coordinates in a higher-dimensional feature space, thereby facilitating linear separation. Multimedia Appendix 2 provides further details about the SVM computation process, including mathematical formulas.

#### Feedforward Neural Network

Biological nervous systems, such as the brain, inspire the information-processing paradigm of FFNN, which is one of the two main types of ANNs [23]. The distinctive feature of this network is the unidirectional flow of information, meaning that the information flow in the model is only in one direction—forward—without any loops or cycles. Information flows from the input nodes through the hidden nodes and to the output nodes.

This network is static and memoryless. Given a data input, FFNN provides a single set of output values instead of a sequence of values. Furthermore, the response produced for an input is independent of the previous state of the network. FFNN automatically learns from examples and uses a backpropagation learning algorithm for determining weights. More details about the FFNN computation process with mathematical foundations are explained in Multimedia Appendix 3.

#### Transformers

Transformers, which dominate natural language processing, have acquired a reputation in computer vision owing to their positive results in many applications such as semantic segmentation, object detection, and image classification. Transformer architecture entirely relies on an attention mechanism to produce global dependencies between input and output, avoiding recurrence. Self-attention assesses the sequence representation by connecting various positions within a single sequence.

In this work, we applied a ViT architecture [24] to neuroimages with very little adjustment, demonstrating better performance in numerous computer-vision tasks. ViT uses a multiheaded self-attention mechanism to catch and learn long-range dependencies between distant positions by averaging attention-weighted positions. This promotes the network’s focus on all of the data of the input sequence. This characteristic encourages us to use ViT for our brain imaging study owing to its capacity to precisely catch interdependencies between spreaded brain regions. More details about the ViT computation process with mathematical foundations are explained in Multimedia Appendix 4.

Nevertheless, the learning dataset is too small, involving substantial data to learn a ViT from scratch. In this regard, we used data augmentation to expand the size of the input data by creating additional data from the original input data. To create new images, we performed some geometric transformations. The visual transformation primarily focuses on translating, flipping random images horizontally, rotating them at 15 angles without cropping, and rescaling the input data to the range of [0, 1].

### Statistical Analysis

We have carried out the performance assessment and the comparison of the classifiers using typical confusion matrix–based evaluation metrics. The confusion matrix has the elements of true positive (TP), false positive (FP), false negative (FN), and true negative (TN). Each column of the matrix indicates an instance of the predicted class, and each row contains a true (correct or actual) class. The following are the metrics used to evaluate the performance of the CAD system.

Sensitivity—also known as recall—is used for calculating the classifier’s ability to correctly predict Alzheimer instances (AD class). On the other hand, the classifier uses specificity to accurately predict all non-Alzheimer instances (HC class) across all inputs.

A classifier should have high sensitivity and specificity. Therefore, the accuracy metric, which calculates the number of correctly classified instances relative to the total number of instances, is the average of these two measures. The precision metric measures the classifier’s ability to quantify the number of TPs of the AD class that receive a correct label in classification.

The combined harmonic mean of both sensitivity and precision gives the *F*_1_-score, which takes a value between 0 and 1. The receiver operating characteristic curve, a method for visualizing a classifier’s ability to diagnose or predict correctly, clearly illustrates the trade-off that arises between the sensitivity and specificity metrics. At various thresholds, the receiver operating characteristic curve plots the TP rate or sensitivity against the FP rate (1 – specificity).

We aim to determine the degree of separability, or the ability to correctly predict class, using the AUROC. The higher the AUROC, the better; 1 would be perfect, and 0.5 would be random. Accuracy, sensitivity, specificity, precision, *F*_1_-score, and AUROC are the six main metrics used to assess the efficacy of each classifier. The following are the mathematical formulas for the first five metrics.


(1) $$Accuracy=\frac{TP+TN}{TP+FP+FN+TP}$$

(2) $$Sensitivity=\frac{TP}{TP+FN}$$

(3) $$Specificity=\frac{TN}{TN+FP}$$

(4) $$Precision=\frac{TP}{TP+FP}$$

(5) $$F1-score=2\frac{Precision\times Sensitivity}{Precision+Sensitivity}$$

## Results

We experimented the performance of the proposed CAD system on patients’ images from the OASIS [26] and ADNI [27] datasets. These datasets contain sMRI and FDG-PET scans along with information about the patients’ demographics and clinical assessments. There are 300 patients for OASIS and 231 patients for ADNI whose age was between 18 and 96 years, and each patient had 3 or 4 accessible PET and T1-weighted MRI scans. Tables1  2 provide more details on the demographic and clinical characteristics of participants.

**Table 1.** The demographic information (gender, race, class, right-handed) of participants.

Variable	OASIS^a^ patients (n=300), n (%)	ADNI^b^ patients (n=231), n (%)
Gender		
Women	80 (26.7)	99 (42.9)
Men	220 (73.3)	132 (57.1)
Race		
Caucasian	174 (58.0)	159 (68.8)
African-American	122 (40.7)	70 (30.3)
Asian	4 (1.3)	2 (0.9)
Class		
Alzheimer	210 (70.0)	149 (64.5)
Healthy	90 (30.0)	82 (35.5)
Right-handed		
Women	77 (96.3)	93 (93.9)
Men	219 (99.5)	130 (98.5)

a OASIS: Open Access Series of Imaging Studies.

b ADNI: Alzheimer’s Disease Neuroimaging Initiative.

**Table 2.** The demographic characteristics and clinical assessment data in terms of age, education, mini-mental state examination, and Alzheimer’s Disease Assessment Scale–Cognitive subscale.

Variable	OASIS^d^ patients, mean (SD; range)	ADNI^e^ patients, mean (SD; range)
Age (years)		
Women	67.78 (43.2‐95.6)	75.3 (5.2)
Men	70.17 (42.5‐91.7)	75.4 (7.1)
Education		
Women	14.3 (1.6; 9-18)	15.6 (3.2)
Men	15.2 (2.7; 8-23)	14.9 (3.4)
Mini-mental state examination^f^		
Baseline (women)	25.4 (0.4; 22-26)	29.0 (1.2; 19-26)
2 years (women)	—^g^	29.0 (1.3)
Baseline (men)	23.8 (1.9; 25-29)	23.8 (1.9; 25–29)
2 years (men)	19.3 (5.6)	29.0 (1.2; 19-26)
Alzheimer’s Disease Assessment Scale–Cognitive subscale^h^		
Baseline (women)	—	7.3 (3.3)
2 years (women)	—	6.3 (3.5)
Baseline (men)	—	7.3 (3.3)
2 years (men)	—	27.3 (11.7)

d OASIS: Open Access Series of Imaging Studies.

e ADNI: Alzheimer’s Disease Neuroimaging Initiative.

f The mini-mental state examination has a possible score range of 0-30.

g Not available.

h The Alzheimer’s Disease Assessment Scale–Cognitive subscale has a possible score range of 0-30.

We used a clinical dementia rating scale to control the dementia status of the dataset; a score of 0 on the scale indicates a normal cognitive level, while a score greater than 0 determines the presence of AD. In this context, we divided the images into 210 (70%) patients with AD and 90 (30%) HCs for the OASIS dataset and 149 (64.5%) patients with AD and 82 (35.5%) HCs for the ADNI dataset. The majority of the samples were identified as men, specifically 220 (73%) for OASIS and 132 (57%) for ADNI, while the majority of the samples were Caucasian, specifically 174 (58%) for OASIS and 159 (69%) for ADNI.

After the preprocessing steps, each slice of sMRI includes 256 × 256 × 176 voxels covering the entire region of the brain with the following parameters: voxel size is 2 × 2 × 2 mm^3^ for ADNI and 2 × 3.1 × 2 mm^3^ for OASIS, isotropic resolution is 1.0 mm, time of repetition is 5050 milliseconds, and time of echo is 10 milliseconds. All slices of reconstructed PET images are resampled to contain 256 × 256 × 207 voxels with a voxel size of 1.2 × 1.2 × 1.2 mm^3^.

The appropriate hyperparameter values for the classifiers were chosen by reviewing prior state-of-the-art work and after doing empirical testing and exploratory analyses. Some of the hyperparameters used in the experiment are presented in Table 3.

**Table 3.** The hyperparameter tuning and classifiers configuration used in the experiment.

Hyperparameter	Search range
Support vector machine	
Multiclass method	One-vs-one (one-vs-all, one-vs-one)
Penality parameter of error	0.001 (0.0001, 0.001, 0.01, 0.1)
Box constraint level	1 (0.001‐1000)
Kernel function	Gaussian (Gaussian, linear, quadratic, cubic)
Kernel scale	2.8
Iteration	30
Standardize data	True
Feedforward neural network	
Number of fully connected layers	1
First layer size	100
Activation	Hyperbolic tangent sigmoid
Learning function	Gradient descent with momentum weight and bias
Iteration limit	1000
Regularizarion strength (λ)	0
Update of weight and bias	Levenberg-Marquardt optimization
Standardize data	True
Vision transformer	
Layers	12
Hidden size D	768
Multilayer perceptron size	3072
Heads	12
Parameters	86 million
Path resolution	16 × 16

For training and testing, 5-fold cross-validation was achieved on each dataset. For each fold, 70% of the data was used for training, 10% for validation, and 20% for testing the effectiveness of each classifier. We conducted experiments on SVM and FFNN using four dimensionality reduction techniques (VAF, LDA, t-SNE, and PCA), as well as on the ViT classifier, without and with data augmentation. During the training process, SVM and FFNN achieved the best results with PCA for the validation data, while the ViT classifier achieved the best results with increased data.

For the test data, we obtained for the OASIS dataset an accuracy of 91.9% (prediction speed ~2000 observations/second, training time 1.5703 seconds) for SVM, 88.2% (prediction speed ~6000 observations/second, training time 7.7715 seconds) for FFNN, and 93.2% (prediction speed ~7000 observations/second, training time 102.3529 seconds) for ViT. The same result was seen for the ADNI data, with an accuracy of 88.6% for SVM (prediction speed ~1300 observations/second, training time 1.4280 seconds), 80.9% for FFNN (prediction speed ~5300 observations/second, training time 8.2319 seconds), and 90.4% for ViT (prediction speed ~7200 observations/second, training time 129.4531 seconds). Tables4  5 provide further details about the top classification results achieved with the proposed ML classifiers for the OASIS and ADNI datasets, respectively, based on six metrics.

**Table 4.** Five-fold cross-validation performance for the Open Access Series of Imaging Studies test data in terms of accuracy, sensitivity, specificity, precision, *F*_1_-score, and area under the receiver operating characteristic curve (AUROC).

Classifier	Accuracy (%)	Sensitivity (%)	Specificity (%)	Precision (%)	*F*_1_-score (%)	AUROC (%)
Support vector machine						
VAF^a^	66.3	61.3	62.1	65.1	52.4	60
LDA^b^	75.6	70.1	69	70.6	68.7	72
t-SNE^c^	80.2	74.5	72.4	71.4	70.1	73
PCA^d^	*91.9* ^*e*^	*86.4*	*90.6*	*87.2*	*89*	*90*
Feedforward neural network						
VAF	62.4	54.1	57.2	51.6	53.4	51
LDA	70.5	66.4	71.4	68.9	72.5	66
t-SNE	72.6	71.3	70.2	69.4	72.8	73
PCA	88.2	85.4	84.6	86.2	83.7	82
Vision transformer						
Without data augmentation	60.8	53.1	54.6	56.8	55.6	61
With data augmentation	*93.2*	*87.2*	*90.5*	*87.6*	*88.7*	*92*

a VAF: voxels-as-features.

b LDA: linear discriminant analysis.

c t-SNE: t-distributed stochastic neighbor embedding*.*

d PCA: principal component analysis.

e Italics indicate the best achieved results.

**Table 5.** Five-fold cross-validation performance for Alzheimer’s Disease Neuroimaging Initiative test data in terms of accuracy, sensitivity, specificity, precision, *F*_1_-score, and area under the receiver operating characteristic curve (AUROC).

Classifier	Accuracy (%)	Sensitivity (%)	Specificity (%)	Precision (%)	*F*_1_-score (%)	AUROC (%)
Support vector machine						
VAF^a^	42.8	59.2	60.4	63.2	50.1	58
LDA^b^	72.1	68.4	67.2	68.4	66.2	70
t-SNE^c^	79.3	71.1	70.1	69.2	68.3	71
PCA^d^	*88.6^e^*	*84.1*	*88.4*	*85.1*	*87.4*	*88*
Feedforward neural network						
VAF	60.9	51.3	56.4	49.1	51	48
LDA	69.1	62.3	70	65.4	70.1	63
t-SNE	70.4	68.1	68.4	67.1	70.4	70
PCA	80.9	84.1	82.3	84.3	81.4	80
Vision transformer						
Without data augmentation	59.3	50.2	51.1	54.4	53.4	57
With data augmentation	*90.4*	*85.4*	*88.6*	*86.9*	*88*	*90*

a VAF: voxels-as-features.

b LDA: linear discriminant analysis.

c t-SNE: t-distributed stochastic neighbor embedding.

d PCA: principal component analysis.

e Italics indicate the best achieved results.

## Discussion

### Main Findings

The main finding is that the development of diagnostic tools applying the ML approach in conjunction with neuroimaging data could substantially help in automating the classification and prediction of AD.

In this context, this study proposed a complete CAD system to successfully classify patients with AD and discriminate them from HC patients. The purpose was to examine the association between SVM, FFNN, and ViT ML classifiers; PCA, LDA, and t-SNE dimensionality reduction techniques; and sMRI and FDG-PET neuroimaging modalities to detect early signs of AD. Furthermore, we aimed to clarify the impact of some data preprocessing strategies, such as noise reduction and data augmentation, on improving the performance of classifiers.

With regard to the sMRI and FDG-PET modalities, they can provide large amounts of information; nevertheless, interpreting all image content is challenging for physicians. The experimental analysis demonstrates that combining these neuroimaging modalities with selected ML classifiers enhances their performance, enabling doctors to provide precise diagnosis and timely patient care. This confirms the theory regarding the benefits of these two modalities. Since sMRI provides high-resolution images of brain anatomical structures, which confirm structural change in the brain, it shows shrinkage of brain tissue and abnormalities, while FDG-PET shows the functionality of the brain.

Regarding the selected dimensional reduction techniques, all of the chosen dimensional reduction techniques performed well as feature extractors when combined with the SVM and FFNN classifiers, but a comparative analysis of the three techniques reveals that PCA outperforms LDA and t-SNE. However, it is important to clarify certain findings: PCA allows the identification of the most significant variables in the data due to its potential to generate new variables, which represent linear combinations of the original variables. Moreover, t-SNE differs from PCA by preserving only small pairwise distances or local similarities, while PCA aims to preserve large pairwise distances to maximize variance. Unlike PCA, LDA is a supervised technique that maximizes class separability in the reduced dimensionality space, thereby retaining the most discriminative features.

Preliminary results from evaluating the complete CAD system using the three classifiers prove that the system is more effective in separating AD and HC classes. The results provided by all the experiments carried out reveal an increase in sensitivity and, consequently, the final accuracy obtained by the basic VAF-SVM model (66.3% for OASIS and 42.8% for ADNI). We compared the performance of the SVM, FFNN, and ViT models using confusion matrix–based metrics.

All models performed well, providing acceptable performance for both databases. Data augmentation/ViT outperformed other models, with accuracies of 93.2% for OASIS and 90.4% for ADNI (see Tables4  5 for more details on results obtained from all models tested on both databases). The second best classifier is PCA/SVM, achieving an accuracy decrease of 1.3% for OASIS and 1.8% for ADNI, compared to the rates obtained by ViT, resulting in overall accuracy rates of 91.9% and 88.6% for OASIS and ADNI, respectively. Therefore, the data augmentation process and the PCA dimensionality reduction method have the potential to impact the overall performance of the ViT and SVM models, respectively.

Moreover, compared to the performance using a single MRI modality, all models performed well using a multimodal MRI/PET environment. The best results with MRI were also obtained with ViT and SVM classifiers. Accuracies of 83.9% for the OASIS dataset and 81.2% for ADNI were obtained using the data augmentation/ViT approach. PCA/SVM achieved accuracies of 82.4% for the OASIS and 80.6% for the ADNI datasets. This draws attention to the potential of integrating multiple modalities to increase the performance of the CAD system.

### Comparison With Prior Work

To verify the convergence of the proposed CAD system, we compared the results obtained with some relevant state-of-the-art ML models. The experimental results show that our models, particularly SVM and ViT, have good performance on both the OASIS and ADNI datasets and achieved better or comparable accuracy to most existing methods in the literature. For the OASIS dataset, the PCA/SVM method had a 91.9% accuracy and the ViT model with data augmentation had a 93.2% accuracy. Nanni et al [33], Khan and Zubair [16], Sethi et al [2], Basheer et al [34], Saratxaga et al [35], and Liu et al [36] got 90.2%, 86.8%, 86.2%, 92.3%, 93%, and 82.6% accuracy, respectively.

The same finding was obtained for the ADNI dataset, where we achieved an accuracy of 88.6% using the PCA/SVM approach and 90.4% using the ViT model by increasing the data. In contrast, the accuracy achieved by Rallabandi et al [37], Jo et al [4], Jo et al [3], Liu et al [36], and Shojaei et al [38] was 75%, 75.02%, 80.8%, 90%, and 87%, respectively. Table 6 compares our best results obtained with the prior state-of-the-art models discussed.

**Table 6.** Comparative study of performance with state-of-the-art machine learning models using the Open Access Series of Imaging Studies (OASIS) and Alzheimer’s Disease Neuroimaging Initiative (ADNI) datasets.

Study	Approach	Dataset	Accuracy	Sensitivity	*F*_1_-score	AUROC^a^
Liu et al [36]	Monte Carlo sampling/ResNet50-CNNs^b^/ensemble classifier	OASIS	82.6	74.3	—^c^	—
Saratxaga et al [35]	ResNet18-based CNNs	OASIS	93	—	—	—
Basheer et al [34]	PCA^d^/ CapsNet-based CNNs	OASIS	92.3	82.3	—	—
Nanni et al [33]	Ensemble of 5 transfer learning models	OASIS	90.2	—	—	—
Khan and Zubair [16]	Chi-square statistical test/RF^e^	OASIS	86.8	80	86.4	87.2
Sethi et al [2]	CNNs/ SVM^f^	OASIS	86.2	**—**	—	—
Our study	PCA/SVM	OASIS	91.9	86.4	89	90
Our study	Data augmentation/ViT^g^	OASIS	*93.2^h^*	*87.2*	*88.7*	*92*
Shojaei et al [38]	Genetic algorithm/3D-CNNs	ADNI	87	—	—	—
Liu et al [36]	Monte Carlo sampling/ResNet50-CNNs/ensemble classifier	ADNI	90	83.5	—	—
Rallabandi et al [37]	FreeSurfer/SVM	ADNI	75	75	72	76
Jo et al [4]	Sliding Window Association Test/CNNs	ADNI	75	—	—	82
Jo et al [3]	Weighted gene coexpression network analysis/RF	ADNI	80.8	—	—	80.8
Our study	PCA/SVM	ADNI	88.6	84.1	87.4	88
Our study	Data augmentation/ViT	ADNI	*90.4*	*85.4*	*88*	*90*

a AUROC: area under the receiver operating characteristic curve.

b CNN: convolutional neural network.

c Not available.

d PCA: principal component analysis.

e RF: random forest.

f SVM: support vector machine.

g ViT: vision transformer.

h Italics indicate the best achieved results.

### Limitations and Future Directions

There are several improvements possible for the proposed CAD system. We aim to enhance the system’s performance by collaborating with more extensive AD datasets and implementing various types of ANN and ML-based classifiers.

The PCA used for feature extraction looks for the principal axis direction, which is used to effectively represent the common features of similar samples. This is very effective for representing the common features of the same kind of data samples, but it is not suitable for distinguishing different sample classes. Therefore, to achieve the purpose of feature extraction, we need to combine PCA with other feature dimensionality reduction algorithms like uniform manifold approximation and projection.

## Supplementary material

10.2196/60866 Multimedia Appendix 1 Principal component analysis.

10.2196/60866 Multimedia Appendix 2 Support vector machines.

10.2196/60866 Multimedia Appendix 3 Feedforward neural network.

10.2196/60866 Multimedia Appendix 4 Vision transformer.

## Abbreviations

AD: Alzheimer disease
ADNI: Alzheimer’s Disease Neuroimaging Initiative
ANN: artificial neural network
AUROC: area under the receiver operating characteristic curve
CAD: computer-aided diagnosis
FDG: fluorodeoxyglucose
FFNN: feedforward neural network
FN: false negative
FP: false positive
HC: healthy control
LDA: linear discriminant analysis
ML: machine learning
MRI: magnetic resonance imagining
N3: nonparametric nonuniform intensity normalization
OASIS: Open Access Series of Imaging Studies
PCA: principal component analysis
PET: positron emission tomography
sMRI: structural magnetic resonance imaging
SPM: statistical parametric mapping
SVM: support vector machine
t-SNE: t-distributed stochastic neighbor embedding
TN: true negative
TP: true positive
VAF: voxels-as-features
ViT: vision transformer
