# Supplementary material for: Improved Alzheimer Disease Diagnosis With a Machine Learning Approach and Neuroimaging: Case Study Development
Source: JMIRx Med. 2025 Apr 21;6:e60866. doi: 10.2196/60866 (PMC12036548; doi:10.2196/60866)
Supplement: Multimedia Appendix 1 [file xmed-v6-e60866-s001.docx]

**Multimedia Appendix 1: PCA**

The PCA computation process with mathematical formulas is explained as follows:

An orthonormal basis vector is generated by the PCA. This vector makes it possible to maximize the dispersion of all projected samples. After the preprocessing steps, the *n* remaining voxels for each subject are rearranged into a vector form. Let $X=\left( x_{ij} \right)_{n\times m}$ be the sample set of these vectors, where *n* is the number of samples (patients), *m* is the number of features and $x_{ij}$ represents the *j*^th^ feature value of the *i*^th^ sample, where *i* =1,2,…,*n* and *j*=1,2,…,*m*. In this research, some gray-scale features are selected as characteristics of the sample. The chosen gray-scale features are: mean, variance, skewness, kurtosis, energy and entropy. The specific steps of PCA are as follows:

1) Standardize the original data by subtracting all samples from the corresponding feature's mean value.

| $\bar{x_{j}}=\frac{1}{n}\sum_{i=1}^{n} x_{ij}$ | (1.1) |
| --- | --- |

2) Calculate the covariance matrix $P=\left( r_{jk} \right)_{m\times m}$, where $r_{jk}$ represents the correlation between the *j*^th^ and *k*^th^ feature.

| $P=\left[ \begin{matrix} r_{11} & \cdots& r_{1m} \\ \vdots& \ddots& \vdots\\ r_{m1} & \cdots& r_{mm} \end{matrix} \right]$ | (1.2) |
| --- | --- |

3) Compute the eigenvalue $\lambda_{i}$ and the eigenvector $e_{i}$ of the covariance matrix *P*:

| $\lambda_{i}e_{i}=Pe_{i}$ | (1.3) |
| --- | --- |

4) Record the resulting eigenvalues in the order of large to small: $\lambda_{1}\geq\lambda_{2}\geq\ldots\geq\lambda_{k}$. Calculate the contribution rate of each principal component.

| $\frac{\lambda_{g}}{\sum_{g=1}^{k} \lambda_{g}}$ | (1.4) |
| --- | --- |

The higher the contribution rate, the stronger the information about the original variables contained in the principal component.

5) Transform the original sample matrix *X* into a new matrix $Y=\left( Y_{ij} \right)_{n\times m_{1}}$, where $i=1,2,\ldots,n$ and $j=1,2,\ldots,m_{1}$.

| $Y=X\times\left[ e_{1},e_{2},\ldots e_{m_{1}} \right]$ | (1.5) |
| --- | --- |

where $\left[ e_{1},e_{2},\ldots e_{m_{1}} \right]$ represents a new feature space composed of $m_{1}$ feature vectors. $m_{1}$ are the principal components extracted by PCA.
