# Supplementary material for: Improved Alzheimer Disease Diagnosis With a Machine Learning Approach and Neuroimaging: Case Study Development
Source: JMIRx Med. 2025 Apr 21;6:e60866. doi: 10.2196/60866 (PMC12036548; doi:10.2196/60866)
Supplement: Multimedia Appendix 2 [file xmed-v6-e60866-s002.docx]

**Multimedia Appendix 2: SVM**

The SVM computation process with mathematical formulas is explained as follows.

SVMs try to maximize the margin between classes (here using the simple linear feature space $x_{i}.x_{j}$), by finding the optimal $\alpha_{i}$ values in the following quadratic programming problem (represented in dual Lagrangian form where *C* is a constant that bounds the misclassification error):

| $max\sum_{i=1}^{N} \alpha_{i}-\frac{1}{2}\sum_{i=1}^{N} \sum_{j=1}^{N} \alpha_{i}\alpha_{j}y_{i}y_{j}\left( x_{i}.x_{j} \right)$  subject to: $0\leq\alpha_{i}\leq C \mathrm{and}\sum_{i=1}^{N} \alpha_{i}y_{i}=0$ | (2.1) |
| --- | --- |

Unlabelled instances are classified using the learned parameters $\alpha_{i}$ and bias $b$, by taking the sign of the following decision function:

| $f\left( x \right)=\sum_{i=1}^{N} \alpha_{i}y_{i}\left( x.x_{i} \right)+b$ | (2.2) |
| --- | --- |
