# Supplementary material for: Improved Alzheimer Disease Diagnosis With a Machine Learning Approach and Neuroimaging: Case Study Development
Source: JMIRx Med. 2025 Apr 21;6:e60866. doi: 10.2196/60866 (PMC12036548; doi:10.2196/60866)
Supplement: Multimedia Appendix 3 [file xmed-v6-e60866-s003.docx]

**Multimedia Appendix 3: FFNN**

The FFNN computation process with mathematical foundations is explained as follows:

*Activation function*

We used the hyperbolic tangent sigmoid, which is a logistic function and ranges from 0 to 1.

| $y_{v_{i}}=2/{\left( 1+exp\left( -2*v_{i} \right) \right)-1}$ | (3.1) |
| --- | --- |

where $y_{i}$ �� is the output of the $i$�th node (neuron) and �� $v_{i}$is the weighted sum of the input connections.

*Training*

Learning occurs by changing connection weights after each piece of data is processed, based on the amount of error in the output compared to the expected result.

We can represent the degree of error in an output node $j$ � in the �$n$ th data point (training example) by :

| $e_{j}\left( n \right)=d_{j}\left( n \right)-y_{j}\left( n \right)$ | (3.2) |
| --- | --- |

��(�)=��(�)−��(�)where ��(�)$d_{j}\left( n \right)$ is the desired target value for $n$ �$nnn$th data point at node �$j$, and ��(�)$y_{j}\left( n \right)$ is the value produced at node �$j$ when the �$n$ th data point is given as an input.

The node weights can then be adjusted based on corrections that minimize the error in the entire output for the �$n$ th data point, given by:

| $\varepsilon\left( n \right)=\frac{1}{2}\sum_{output node j} e_{j}^{2}\left( n \right)$ | (3.3) |
| --- | --- |

To change the hidden layer weights, the output layer weights change according to the derivative of the activation function (backpropagation of the activation function).
