# Supplementary material for: Improved Alzheimer Disease Diagnosis With a Machine Learning Approach and Neuroimaging: Case Study Development
Source: JMIRx Med. 2025 Apr 21;6:e60866. doi: 10.2196/60866 (PMC12036548; doi:10.2196/60866)
Supplement: Multimedia Appendix 4 [file xmed-v6-e60866-s004.docx]

**Multimedia Appendix 4: ViT**

The ViT computation process with mathematical foundations is explained as follows:

We divided each input image (of dimensions$H\times W\times C$) to a grid (of dimension ($H/P$) × ($W/P$)) containing a set of non-overlapped patches having a fixed size. Each of these patches functions as a token and contains a fixed number of pixels (16×16 pixels). Where *H*, is the height, *W* is the width, and *C* is the number of channels.

After that, the patches are transformed into a 1D embedding vectors sequence. After flattening, the image transforms to a token sequence, $x\in\mathbb{R}^{H\times W\times C}$. The image is flattened into a sequence of 2D patches $x_{p}\in\mathbb{R}^{CP^{2}\times N}$. Where (*H*, *W*) is the image size, (*P,* *P*) is the size of each image patch, and $N={HW}/{P^{2}}$ is the number of patches.

The flattened patches are then linearly projected onto lower dimensional vectors (token embeddings). Token embeddings allow to save patch content. So that each patch is vectorized and linearly projected into tokens by:

| $\hat{X}=\left[ x_{class}, x_{1}E, x_{2}E,\ldots,x_{N}E \right], E\in\mathbb{R}^{CP^{2}\times D}$ | (4.1) |
| --- | --- |

To provide spatial information, the token embeddings are augmented with learnable positional embeddings. These embeddings encode each token's relative position in the sequence. A positional embedding known as $E_{pos}$ is added to the token as follows:

Token embeddings are complemented by learnable positional embeddings $E_{pos}$ to supply spatial information. These latter integrations make it possible to encode the relative position of each token in the sequence.

| $Z_{0}= \hat{X}+E_{pos}, E_{pos}\in\mathbb{R}^{\left( N+1 \right)\times D}$ | (4.2) |
| --- | --- |

The vectors are then passed via a series of transformer blocks. Each transformer block has two sub-layers: a multi-head self-attention (MSA) layer and a feed-forward layer (MLP) with Layer-Norm (LN).

Next, the vectors flow through a series of transformer blocks that each contains a feed-forward layer (MLP) with layer-norm (LN) and a multi-head self-attention (MSA) layer.

At the end, at the output of the transformer blocks, we apply a classification head represented by a linear layer generating the probabilities of the input image class.
